# Fermentation of Biomass and Residues from Brazilian Agriculture for 2G Bioethanol Production

**DOI:** 10.1021/acsomega.4c06579

**Published:** 2024-08-23

**Authors:** Douglas José Faria, Anna Paula Azevedo de Carvalho, Carlos Adam Conte-Junior

**Affiliations:** † Department of Biochemistry, Chemistry Institute, Federal University of Rio de Janeiro, Rio de Janeiro, RJ 21941909, Brazil; ‡ Research Support Group on Nanomaterials, Polymers, and Interaction with Biosystems (BioNano), Chemistry Institute, Federal University of Rio de Janeiro, Rio de Janeiro, RJ 21941909, Brazil; § Center for Food Analysis (NAL), Technological Development Support Laboratory (LADETEC), Federal University of Rio de Janeiro, Rio de Janeiro, RJ 21941598, Brazil; ∥ Graduate Program in Chemistry (PGQu), Chemistry Institute, Federal University of Rio de Janeiro, Rio de Janeiro, RJ 21941909, Brazil

## Abstract

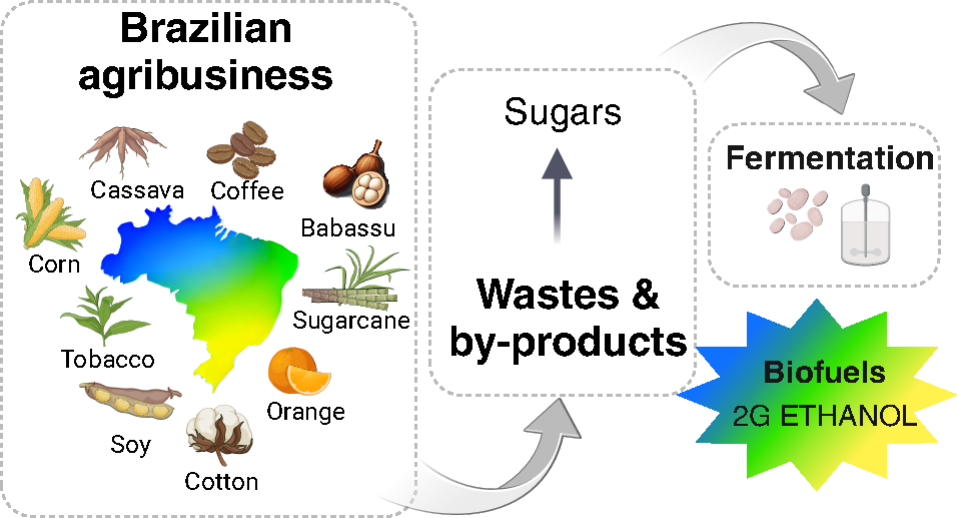

Brazil is one of the world’s leading producers of staple foods and bioethanol. Lignocellulosic residual sources have been proposed as a promising feedstock for 2G bioethanol and to reduce competition between food and fuels. This work aims to discuss residual biomass from Brazilian agriculture as lignocellulosic feedstock for 2G bioethanol production as bagasse, stalk, stem, and peels, using biorefining concepts to increase ethanol yields. Herein, we focused on biomass chemical characteristics, pretreatment, microorganisms, and optimization of process parameters that define ethanol yields for bench-scale fermentation. Although several techniques, such as carbon capture, linking enzymes to supports, and a consortium of microorganisms, emerge as future alternatives in bioethanol synthesis, these technologies entail necessary optimization efforts before commercial availability. Overcoming these challenges is essential to linking technological innovation to synthesizing environmentally friendly fuels and searching other biomass wastes for 2G bioethanol to increase the biofuel industry’s potential. Thus, this work is the first to discuss underutilized lignocellulosic feedstock from other agrifoods beyond sugar cane or corn, such as babassu, tobacco, cassava, orange, cotton, soybean, potatoes, and rice. Residual biomasses combined with optimized pretreatment and mixed fermentation increase hydrolysis efficiency, fermentation, and purification. Therefore, more than a product with a high added value, bioethanol synthesis from Brazilian residual biomass prevents waste production.

## 1 Introduction

Brazil is essentially self-sufficient in food commodities, with many crops grown and harvested to produce food or livestock (i.e., food and feed crops, fiber and oil crops, and industrial crops).^1^ Brazil’s 2022/23 crops are expected to reach up to 312.4 million tons of soy, cotton, corn, rice, and other products in national grain production.^2,3^ Moreover, Brazil has emerged as a global supplier of a wide range of crops and basic foodstuffs such as soybean, soybean derivatives, corn, herbal cotton, sugar cane, coffee, cassava, citrus, cacao, grains, and ethanol since the early 21st century.^3^ Moreover, Brazilian sugar cane ethanol production started in the 1970s due to the oil crisis in search of alternative fuels.^4−6^ In 2019, more than 45% of the energy power supply in Brazil came from renewable sources.^7^ Biofuels represent 25% of transport fuels in Brazil, with the highest presence of bioethanol representing 49% of the energy of combined gasoline and ethanol.^7^ Although sugar cane^8,9^ and corn^10−12^ are major conventional crops used in first-generation (1G) bioethanol, such crops cannot achieve world demand for bioethanol production due to their primary value as food and feed crops.^9,11,13^ Therefore, since 2010 several Brazilian institutions and research groups have described efforts toward cellulose-based sources as agroindustrial wastes as promising feedstock for bioethanol production,^14^ once cellulose is the most abundant agroindustrial biomass residue available.^15,16^

Regarding biomass residues, Brazil has different sources of agrifoods generating biomass residues, such as sugar cane bagasse, rice straw, and corn cob/corn leaf.^14^ All of these energy sources are considered industrial waste due to their application in the synthesis of high-value-added products. Therefore, applying such residual biomasses on a bioethanol industrial and commercial scale is a challenge.^17^

Bioethanol is one of the most promising fuels on the market, partially capable of replacing fossil fuels, requiring 68% less energy production than high-octane gasoline.^18,19^ The production commonly uses sugar cane, sweet potato, beetroot, and cereals, competing with the food industry. Thus, other synthesis methods have been studied, such as hydrolyzing bagasse to sugars and the use of residues in the fermentation process.^19−22^ Therefore, novel and efficient bioprocesses for underutilized biomass are at the forefront of biotechnological research and industrial application.

The residues for bioethanol production require a high amount of carbohydrates, microorganisms with good viability for propagation, and specific enzymes to break these carbohydrates into simpler molecules.^20,23^ Brazil presents a high amount of food waste and microorganisms that open many possibilities for optimizing the fermentation to generate industrial bioethanol.^24^ Therefore, beyond the product synthesis with high added value, biomasses residual waste may not be discarded.^21,25−28^ The synthesis occurs through the fermentation reaction of sugars converted into ethanol. Fermentation depends on several factors such as time, temperature, specificity of metabolites produced by microorganisms, pH, and contaminants.^26,27,29^

After pretreatment, the residue undergoes hydrolysis by chemical or biological routes. Chemical hydrolysis can be achieved by diluted or concentrated sulfuric acid.^29,30^ The thermal stability and specificity of enzymes such as amylases, cellulases, pectinases, and glycosidases^29,31^ influence enzymatic hydrolysis. The use of enzymes is encouraged by milder temperature and pH conditions and lower consumption of water vapor, making the process more industrially applicable.^20,32^ Furthermore, a recent bibliometric analysis showed higher efforts over the last 10 years.^33^*Saccharomyces cerevisiae* is a microorganism commonly used in lignocellulosic hydrolysates^34^ for fermentation, a process that occurs at temperatures of 30 °C until the ethanol concentration is >10% in the fermentation broth. Other microorganisms such as *Xanthomonas axonopodis*, *Candida parapsilosis*, and *Trichoderma harzianum* can also be cultivated and used in fermentation for producing cellulase enzymes that break down carbohydrates, generating bioethanol with purity higher than 97.5%.^35,36^

Once Brazil alone was the most significant sugar cane producer, sugar cane bagasse^37−40^ was extensively reviewed and proposed as potential agricultural waste in second-generation (2G) bioethanol due to high contents of fermentable sugars in biomasses (cellulose, lignin, and hemicellulose). However, the offer of bagasse and straw for bioethanol production on an industrial scale has also been prospected to be insufficient, demanding alternative lignocellulosic materials with potential in 2G ethanol production.^40^ Recently, researchers overviewed literature to design and propose an integrated soybean–sugar cane biorefinery with a high potential for implementation in Brazil.^41^ However, since 1998, the increased possibility of other potential Brazilian agrifoods (i.e., babassu mesocarp) was prospected to produce low-cost ethanol compared to sugar cane or conventional starch-rich material. The study showed the net profitability of ethanol production of 40% for babassu coconut as opposed to 10% for sugar cane.^42^ Babassu wastes received very little attention in bioethanol production after two decades despite its feasibility, high essential density, and suitable lignin content in babassu kernel nut for sustainable bioenergy production later commented on.^43^

Brazil has several biomasses that could be applied to the synthesis of 1G bioethanol, such as papaya, banana, babassu, and potato, which compete with the food industry. On the other hand, the third-generation (3G) ethanol, obtained from algae, stood out by its greater photosynthetic efficiency and less competition for agricultural resources, but its production on a commercial scale still requires more technological advances. In that sense, the residues of those crops, such as banana peel, coffee peel, potato peel, papaya peel, corn cob, and dry corn leaf peels, among countless residues that are commonly discarded, can be used for 2G bioethanol synthesis. Therefore, it provides a reagent not used by the food industry to synthesize a high-added value product for a greener future: 2G bioethanol, which has potential as a clean and renewable transportation fuel. Moreover, by blending it with gasoline, 2G bioethanol reduces conventional fuels’ carbon footprint and allows countries to meet stringent emission standards.

Although several reports are available on review analysis of bioethanol production from biomasses, they focused on sugar cane bagasse,^9,14,44^ corn residues,^10,13^ or food wastes rich in carbohydrates.^45^ Moreover, until now, although some assessment on other lignocellulosic^46^ was performed, some of the critical factors have not yet been discussed, such as (i) reaction conditions at pretreatment of biomass waste to maximize bioethanol conversion efficiency; (ii) yields obtained by the alcoholic fermentation process; and (iii) the impact on current Brazilian fuel production. Therefore, this Review focuses on recent studies with different biomass residues available in Brazil for 2G ethanol. Besides, the analysis of 1G and 2G ethanol in different regions of Brazil using sugar cane and processing residues to increase its industrial production leads to an economic improvement and good environmental impact in Brazil and the global energy matrix.

Once the use of agricultural wastes to produce bioethanol does not compromise food security, contributes to waste management, prevents environmental degradation, and ensures energy security, this work can potentially impact not only locally but globally, improving manufacturing activities, enhancing farming and other food production related to agricultural waste generation, renewable fuel consumption, and emission of toxic gases.

## 2 Agrifood Residual Biomasses and Wastes

**Figure 1:**
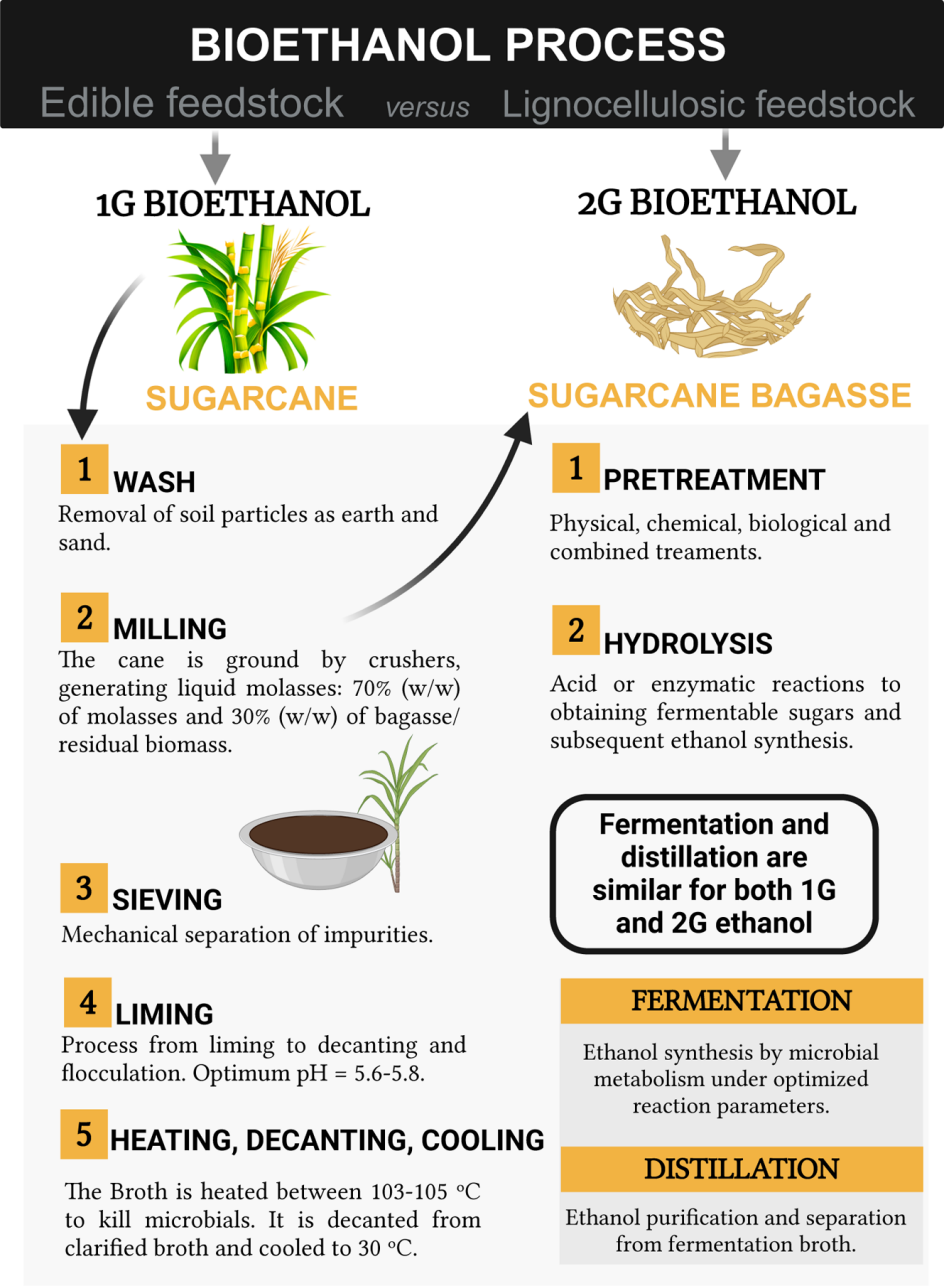
Lignocellulosic feedstocks (2G bioethanol) versus edible feedstock (1G bioethanol) using sugar cane as an example of the bioethanol process. While industrial synthesis of 1G bioethanol occurs mainly through sugar cane molasses, 2G bioethanol can be made by residual biomasses such as sugar cane bagasse, corn cobs, potato husks, and rice husks.

Bioethanol can be made from different cellulosic biomasses, which can be classified into three types: (i) containing sucrose (sugar cane, beet); (ii) starchy compounds (babassu, rice, wheat); and (iii) cellulosic biomass (wood, straw). This process includes several essential steps for excellent fermentation performance, as shown in Figure 1.^47−49^ Finally, bioethanol produced during the fermentation process is purified. Distillation of fermentation broth provides greater product purity.^47^

The lignocellulosic materials used in bioethanol synthesis undergo pretreatment and hydrolysis to release simpler sugars, such as d-glucose, d-xylose, and l-arabinose. However, the pretreatment stage generates high concentrations of sugars and inhibitors of cell growth (i.e., formic acid, acetic acid, furfural commonly found in hemicellulose hydrolysates) that can negatively influence fermentation. Among the inhibitors, the high stress for the microorganisms, temperature, and the possibility of contamination that would generate a dispute for the substrate inside the fermenter can be aggravating factors that may hinder the reaction success.^50^ If fermentation at high temperatures leads to higher bioethanol synthesis yields, strains with a tolerance to thermal stress should be considered.

Bioethanol production by Simultaneous Saccharification and Fermentation (SSF) employs temperatures above 40 °C for enzymatic saccharification. In addition, higher temperatures can help to reduce the number of contaminants that may appear during the process. Therefore, knowing how to deal with microbiological growth and, consequently, the release of enzymes that help in fermentation is a critical factor for the success of fermentation and bioethanol production. The higher microorganism growth related to reaction adversities (stress, temperature, agitation, contaminates, etc.) enhances the bioethanol yield and purification, generating a higher quality product. A technique used to find microorganisms that adapt to these requirements is the selection of species with the same diversity, such as places with warmer climates and abrupt temperature increases.^51,52^

Several lignocellulosic residues can be used for fermentation, such as banana peel and peduncle; rice straw and husk; sugar cane straw and bagasse; soybean hulls and *okara*; corn stover, straw, cob, and stalk; cotton spinning, stem, and stalk; cassava stem and peels; coffee cut stems and husks; wheat straw; tobacco stalk; and reed straw. Palacios et al. (2017)^53^ evaluated the use of sulfuric acid as a pretreatment of banana peel, reaching hydrolysis of ∼99% after 48 h; thus, the fermentation of the hydrolysate using *Kluyveromyces marxianus* produces a bioethanol yield of 21 g/L within 24 h, indicating an excellent resource for biofuel. Danmaliki et al. (2016)^54^ used an alkaline treatment to hydrolyze the banana peel and then used *S. cerevisiae* for fermentation, reaching 80 ppm of bioethanol concentration after 48 h.

Chen et al. (2021)^55^ evaluated the use of waste straw for bioethanol synthesis, reaching 37.0 g/L concentration by subcritical water pretreatment combined with a separate high solid. Lyu et al. (2021)^56^ used hydrothermal pretreatment in whole-plant cassava by the SSF method, reaching a bioethanol yield of 53.9% and confirming the efficiency of the production process through economic and environmental studies. Wheat and rye stillages are biomasses that underwent diluted acid pretreatment, followed by 48 h of fermentation using *S. cerevisiae*, providing 20.0 g/L of bioethanol.^57^ Using biological pretreatment with *Spathaspora passalidarum* U1-58 in two types of fermentation (SSF and SHF) for 96 h generated 42.6–53.25 g/L of bioethanol using corn cob.^58^

**Figure 2:**
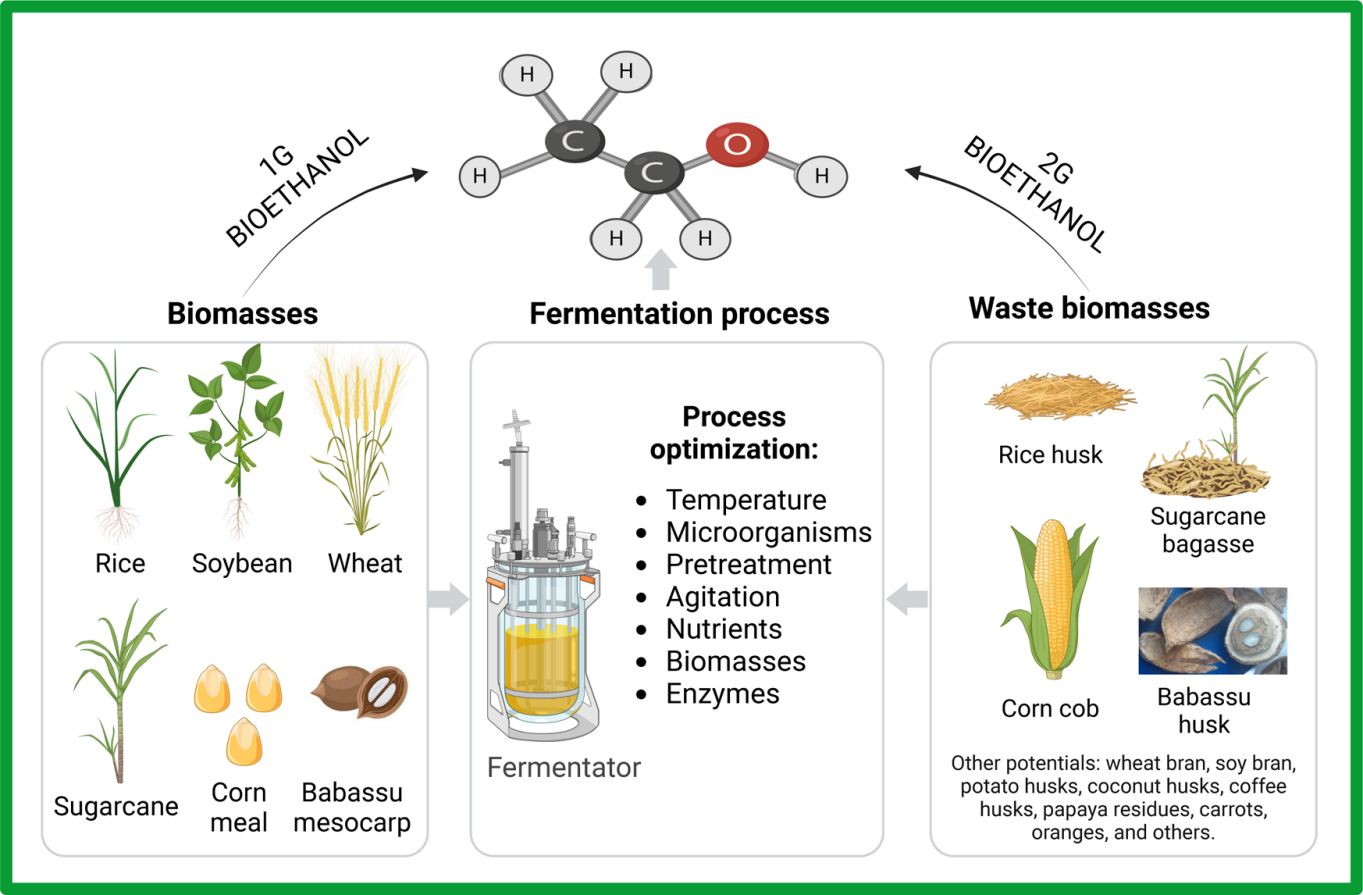
Examples of Brazilian biomass and biomass residues as feedstock for bioethanol fermentation.

Another biomass of great importance in the synthesis of biofuels is babassu (*Orbygnia phalerata*), widely cultivated in Brazil and with several applications in the food, detergent, and cosmetics industries. Besides the usefulness of babassu oil for biodiesel production, the residues generated during oil extraction may be used in bioethanol synthesis by microbial fermentation.^59^ The oil extraction process generates a cake composed mainly of protein (23%), carbohydrates (62%), and lipid residues (4.5%), while babassu mesocarp flour, obtained from the mechanical crushing of the coconut mesocarp, contains approximately 50% (w/m) starch and 10% (w/m) fiber on a wet basis.^51,59^ The composition of babassu cake makes it an excellent raw material for the SSF method, especially for the cultivation of filamentous fungi, enabling the production of complex multienzymes containing amylases, xylanases, cellulases, and proteases, which are capable of hydrolyzing starch granules crude.^60−62^ In addition, babassu mesocarp flour generated during babassu oil extraction can be used as a base for fermentation due to the large amount of starch in its composition.^51,60,62^ The high number of celluloses and hemicelluloses in the bark residue favors its use in bioethanol synthesis, from the fermentation of lignocellulosic biomass using specific microorganisms.^51,52,62−64^ Thus, babassu coconut mesocarp is a promising resource to produce ethanol, thin film for application in electrochemical sensors, and bioactive film, presenting great versatility for its technification and favoring the creation of local industries.^60,61,63,64^Figure 2 shows examples of raw biomass and biomass residues that can be used to synthesize bioethanol from fermentation.

## 3 Pretreatment of Residues for Bioethanol Production

The pretreatment step plays a crucial role in cellulose conversion. The compact structure of lignocellulose is disrupted to expose the cellulose fiber. Thus, changing the cellulosic biomass’s structure will improve the cellulose availability for enzymes to convert carbohydrate polymers into fermentable sugars.^65^ Several pretreatments can be used to reduce the lignin content and facilitate enzymatic hydrolysis performed by cellulase enzymes. Such techniques are based on mechanical, physical, chemical, biological, or combination processes, which depend on the degree of separation required and the purpose.

### 3.1 Physical Treatments

Several physical methods exist to treat biomass: milling, grinding, ultrasound, microwaves, and radiation.^66^ A substantial number of physical processes are under development and commonly require specific equipment for each type of technology. Several reviews have been published in recent years describing the modes of action, devices, and main effects on raw materials of milling methods as biomass processing techniques.^67^

### 3.2 Chemical Treatments to Increase Hydrolysis

Chemical pretreatments mainly include hydrolysis acid at different temperatures and concentrations.^68^ Concentrated acids allow the hydrolysis of hemicelluloses and cellulose, whereas diluted acids make the hydrolysis more specific for hemicelluloses. Alkaline treatment has also been used and includes the aid of sodium, potassium, calcium hydroxides, or ammonia.^68−71^ Delignified corn cob^72^ and corn straw^73^ were highly competitive with corn meal as feedstock for ethanol fermentation. Delignified corn cob residue provided an ethanol titer and yield of 75.07 g/L and 89.38%, respectively.^72^

Hydrochloric acid, sulfuric acid, formic acid, and acetic acid enhance the cellulose removal efficiency. Ionic liquids, organic solvents, and deep eutectic solvents are also being studied as more environmentally friendly than other chemicals.^74,75^Table 1 displays studies using chemical methods for the hydrolysis of biomass.

**Table 1:** Pretreatment Technologies Used in Residues Generated from Agricultural Activities for Bioethanol Production

Physical Treatment				
milling	biomass	variables	main results	ref
knife	wheat straw	mesh size of the screen number and position of the knives	use of large particle size may be advantageous over small sizes	Yang et al. 2023^80^
wet-disk	sugar cane bagasse	solid/liquid ratio	substantial increase in ethanol production	Wang et al. 2018^81^

Chemical Treatment				
hydrolysis	biomass	conditions	results	ref
acid				
2% H_2_SO_4_	cornstalk	121 °C, 60 min	effectiveness in removing hemicellulose	Li et al. 2016^82^
organosolv				
ethanol 50%	wheat straw	180 °C, 40 min	89.2% cellulose conversion	Salapa et al. 2017^83^
ethanol 50%	sugar cane bagasse	190 °C, 100 min	cellulose-rich solid (65.6% cellulose, 7.9% hemicellulose, and 18.5% lignin)	Espirito Santo et al. 2018^79^
ionic liquid				
cholinium lysinate ([Ch][Lys])	sorghum bagasse	140 °C, 60 min	glucose yield: 88%	Pérez-Pimienta et al. 2021^84^
triethylammonium hydrogen sulfate ([TEA][HSO4])	widely grown perennial grass	140 °C, 50 min	digestibility of biomass enhanced from 16.7% to 84.1%	Poolakkalody et al. 2023^85^
deep eutectic solvent				
ChCl-lactic acid	eucalyptus	110 °C, 6 h	93% of lignin and 95% of xylan were removed	Shen et al. 2019^77^
ChCl-*p*-hydroxybenzoic acid (PB)	woody biomass	160 °C, 3 h	69% of lignin could be removed	Wang et al. 2020^76^

For the effectiveness of chemical treatment, solvent charges are applied in high proportions in batch systems with greater hydrolysis yields. Therefore, despite numerous techniques, the amount of waste generated using acids and solvents makes the process disadvantageous from an environmental point of view. Thus, biological methods are an excellent alternative to break the stigma of balancing high industrial economic yields with reducing environmental impacts.^76−79^

### 3.3 Biological Treatments and Advantages

Biological methods such as fungi, bacteria, microbiological consortia, and enzymes are also excellent techniques to remove lignin.^86−88^ Studies related to consortiums of microorganisms show an increase in the release of extracellular enzymes as the consortium species grow in an environment that favors competition for nutrients.^36,89^ Thus, such measures can be applied to improve the hydrolysis of lignocellulosic material.^36^ Compared to the other pretreatments, using microorganisms requires less energy due to the mild temperature conditions and causes less environmental pollution due to the absence of toxic solvents. However, microorganisms make the process slower, decreasing the hydrolysis efficiency.^63,89−94^ Other pretreatment processes evaluated in recent years use CO_2_ and the combination of microorganisms or enzymes to increase substrate specificity and, simultaneously, make the method cleaner, with a decrease in energy and greenhouse gas consumption.^95−98^

### 3.4 Supercritical Carbon Dioxide (scCO_2_)

The use of scCO_2_ is widely considered for biomass treatment processes due to the parameters used for the hydrolysis of lignocellulosic material, such as milder temperatures and pressures (∼31 °C and 7.3 MPa).^99,100^ Thus, there is a chance to explore CO_2_ as a pretreatment for recovering bioproducts in sustainable processes. scCO_2_ is a cheap, environmentally friendly, and easy-to-recover solvent. Besides, it has low viscosity and low surface tension, which is helpful for many processes due to the more accessible surface contact.^99−105^ Some studies indicate that water in biomass, when combined with scCO_2_, produces a mixture of carbonic acid, generating a weak acid in the environment, promoting hydrolysis and intensifying the mass transfer.^106^ The most significant advantage of scCO2 is the physical pretreatment without loss of biomass hydrolysis efficiency considering temperature and pressure parameters.^99,100,102−105,107^ Therefore, scCO2 is a technique that needs optimization and analysis given the different biomasses that can be hydrolyzed by this method. Despite this, it is effective and has excellent potential for future industrial use. Finally, methods such as alkaline grinding, alkaline fungus, steam explosion, milling, hot water, metal activation, and mechanical salt methods can be combined to improve hydrolysis.^108−112^

### 3.5 Combined Pretreatments

The use of combined pretreatments is a common technique used in several works to induce an increase in the hydrolysis yield and, consequently, facilitate the fermentation. Mihiretu et al. (2017)^113^ evaluated microwave-assisted pressurized hot water to treat wood waste. This technique improved the dissolution of hemicelluloses in a shorter time. The sugars contained about 90% oligomers and 5% monomeric xylose. Therefore, the combination of methods allowed the establishment of a more economical and effective technique. However, despite providing cost efficiency, there is a greater need for energy application due to the combination of two physical processes.

Microwave hydrothermal treatment (MH), fungal pretreatment (PC), and its combination (MH+PC and PC+MH) were evaluated in the treatment of residual grains and increased the yield of reducing sugar and sucrose of the biomass residues. In addition to the “combination” factor, temperature, power, and time^114^ are also crucial. The hydrolysis step can also be more efficient with the use of milling (physical treatment) mixed with P_2_O_5_ (chemical treatment), generating glucose with high yield from residual biomass, making the quality of the method sufficient for the synthesis of bioethanol by fermentation.^115^

Ammonia: Salt solvent-based treatments showed ∼85% lignin dissolution while reducing cellulose crystallinity.^116^ In this way, the excellent digestibility makes the consumption of enzymes 50% lower than that of conventionally treated cellulose. Integration of different treatments can facilitate the release of sugar and increase alcoholic fermentation.^116,117^

Besides that, the yield of reducing sugars from the hydrolysis process with combined treatment reached 266.5 mg/g of rice husk, higher than untreated rice husk hydrolysate (72.67 mg/g of rice husk rice).^118^ Recently, urea (4.87% content) and stem explosion (1.22 MPa pressure) were combined to pretreat corn stover, enhancing enzymatic saccharification and providing an ethanol fermentation sugar alcohol conversion rate of 48.30%.^119^ These results indicated that the combined pretreatment of dilute sulfuric acid and steam explosion significantly increased the yield of reducing sugars and subsequent fermentation.

**Figure 3:**
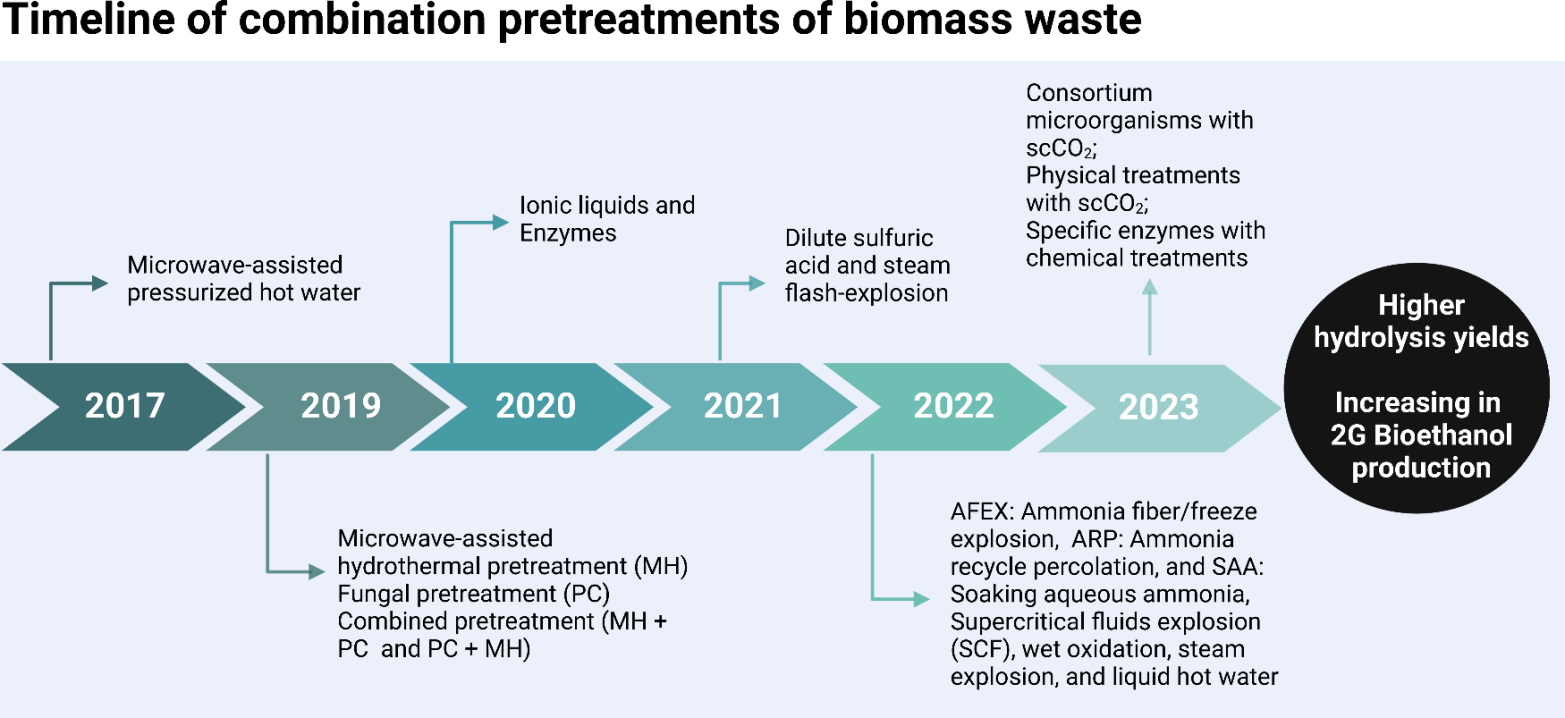
Timeline reports on combined treatments with increased yields of biomass hydrolysis and possible combinations that can be analyzed in the future using enzymes and scCO_2_

All of the cited works show good results in combining pretreatments before fermentation. Numerous methods can be analyzed and used in different biomass residues. Therefore, the feasibility of using scCO_2_ combined with physical, chemical, and biological treatments improves the hydrolysis yields and increases the production of 2G bioethanol. Figure 3 shows combined treatments with increasing biomass hydrolysis and other potential combinations that can be analyzed by using enzymes and scCO_2_.

## 4 Fermentation Process in 2G Bioethanol Synthesis

**Figure 4:**
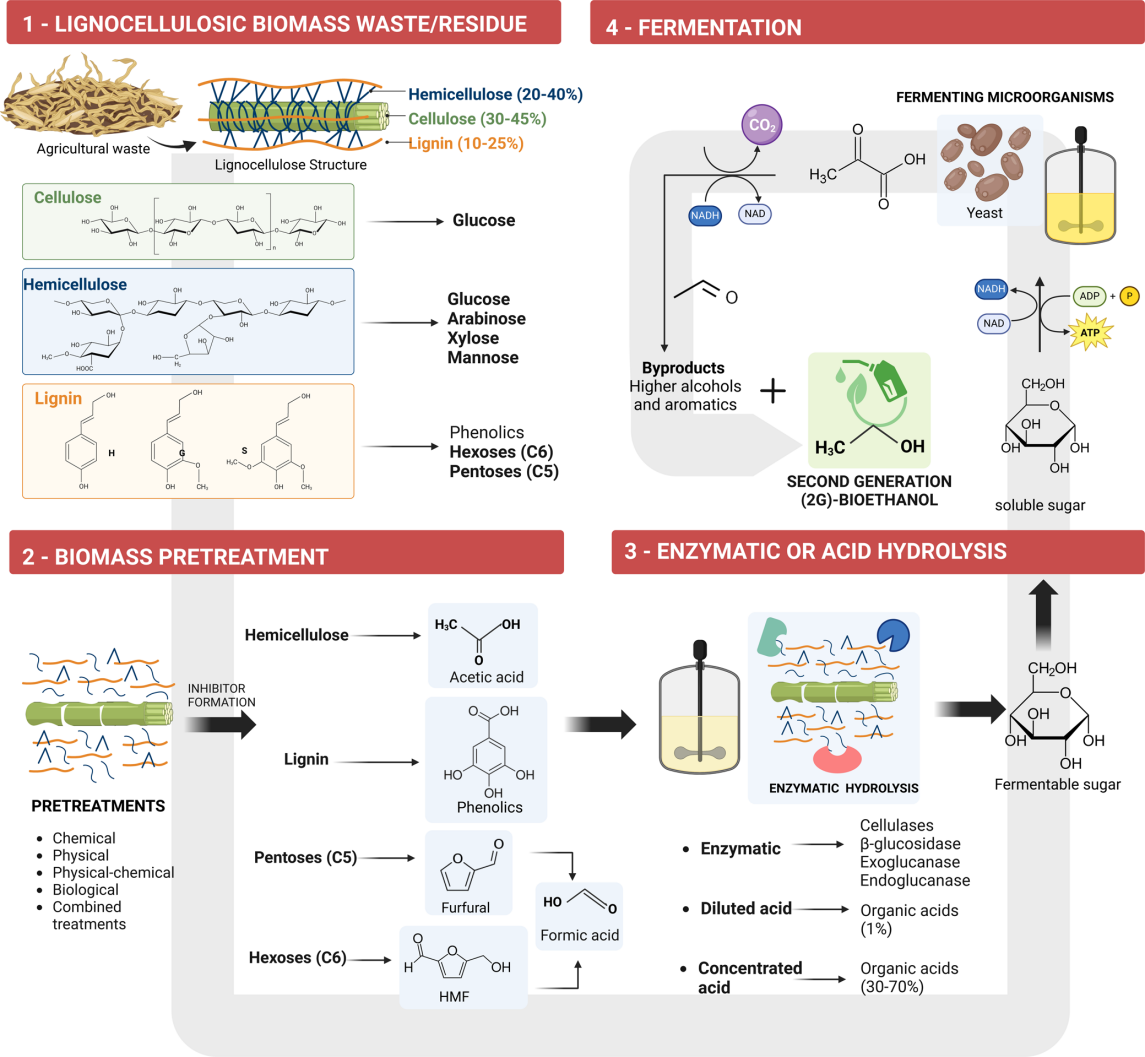
Bioethanol production from lignocellulosic biomass residues/wastes: (1) Composition of lignocellulosic biomass residue/waste. (2) Inhibitor formation effect of the pretreatment step and main inhibitory compounds formed. (3) Hydrolysis of pretreated lignocellulosic residue material. (4) Fermentation by microorganisms to convert soluble sugars (generated in hydrolysis) into 2G bioethanol and byproducts.

The biochemical conversion of lignocellulosic biomass into bioethanol^68,120^ can occur by different microbial fermentation processes (Figure 4). In separate hydrolysis and fermentation (S-HF), enzymatic hydrolysis and fermentation are performed sequentially. In S-HF, the enzymatic saccharification of starchy biomass or pretreated lignocellulosic biomass is performed first at the optimal temperature of the saccharifying enzyme. On the other hand, in simultaneous saccharification and fermentation (SSF), hydrolysis and fermentation coincide in the same reactor, reducing initial costs. The inhibition of enzymes by sugar is eliminated once sugars are immediately converted to alcohol by fermenting microorganisms.^121^ The SSF stood out by lower energy consumption, higher ethanol yield, reduced process time, and lower contamination risks since only a single reactor is used. During ethanol synthesis through lignocellulosic materials, the alcohol concentration is essential once it affects the purification costs and the inhibition of microorganisms. In the simultaneous saccharification and cofermentation (SSCF), the viscosity of the medium is kept by feeding new substrates in the reactor, decreasing the microbiological stress and increasing the amount of substrate available for fermentation.^121^ On the other hand, the continuous insertion of substrates or other nutrients can favor contamination inside a reactor, causing a decrease in bioethanol yield. Table 2 shows examples of ethanol concentrations formed from different fermentation processes and how SSF-type fermentation provides superior results.

**Table 2:** Brief Overview of 2G Ethanol Production by Biomass Wastes, Residues, or Coproducts Commonly Generated in Brazilian Agribusiness^a^

	cellulose conversion, hydrolysis		fermentation		recovery	
feedstock (residue/waste)	biomass pretreatment	sugar yield/conversion efficiency	process	culture medium	ethanol titer, yields, or efficiency	ref
babassu mesocarp starch	mechanical processing	–	S-HF	*Saccharomyces cerevisiae*	290 L/ton	Baruque Filho et al. 1998^42^
sweet sorghum bagasse	diluted sulfuric acid solution	–	SSF	*S. cerevisiae* ATCC	38 g/L, 89.4%	Wang et al. 2013^132^
tobacco stalk	alkaline (7% w/w NaOH, 120 °C, 1 h)	413 kg/ton glucose	S-HF	*S. cerevisiae*	275 kg/ton, 72.7%	Yuan et al. 2019^133^
		225 kg/ton xylan				
	acid-catalyzed steam (3% w/w SO_2_, 180 °C, 15 min)	408 kg/ton glucose			269 kg/ton, 70.6%	
		227 kg/ton xylan				
tobacco product waste	alkaline (10% w/v NaOH, 80 °C, 3 h)	91.3%	S-HF	*Mucor hiemalis*	44.3 L/ton, 97%	Sarbishei et al. 2021^134^
cigarette butts	deacetylation and lipase treatment	12 g/L, 70% w/w	S-HF	*S. cerevisiae* EC 1118	1.53 g/L, 12.73%	Battista et al. 2023^135^
				*Lachancea fermentati* LS16	2.44 g/L, 20.29%	
				*Metschnikowia pulcherrima* MALV5	2.14 g/L, 17.80%	
coffee residue waste	popping (1.47 MPa, 10 min)	34.4 g/L, 85.6%	SSF, enzymatic hydrolysis	*S. cerevisiae*	15.3 g/L, 87.2%	Choi et al. 2012^136^
coffee cut-stems	simulation:	hemicellulose conversion: 100%, cellulose conversion: 21.6%.	enzymatic hydrolysis, fermentation	*Zymomonas mobiliz* ZM4 (pZB5)	240 L/ton	Triana et al. 2011^137^
	liquid hot water (220 °C, 30 min)					
coffee residue waste	ethanol 60%, 150 °C, 2 h	d-mannose: 16 g DW, 22.7% of total sugars	enzymatic hydrolysis, fermentation	*S. cerevisiae*	11.3 g DW, 93% DW	Nguyen et al. 2017^138^
sticky coffee husks	–	total sugars: 28.7%	batch fermentation	*S. cerevisiae*	13.6 g/L, 92%	Gouvea et al. 2009^139^
cassava stems	thermohydrolysis	10.35 g/L	enzymatic hydrolysis, fermentation	*Rhizopus* spp.	5.27 g/100 g	Kouteu Nanssou et al. 2016^140^
cassava peels		10.50 g/L			2.6 g/100 g	
tapioca agrowastes (cassava)	delignification: 10% NaOH, 160 °C, 30 min	0.79 g/g dry cassava peels and 0.68 g/g *onggok*	S-HF; reflux column distillation	*S. cerevisiae* IPA1	>92%	Amalia et al. 2021^141^
cotton spinning (hard wastes)	delignification: 12% NaOH, 5 °C, 3 h	82% hydrolysis efficiency	enzymatic digestibility, S-HF	*S. cerevisiae*	2.5 g/L, 65%	Ranjithkumar et al. 2022^142^
cotton stalk	delignification: 3% NaOH, T_room_, 24 h	29.40 g/L sugars, 96.5%	two-stage dilute acid hydrolysis, cofermentation	*S. cerevisiae* VS3 and *Pichia stipitis* NCIM3498	11.64 g/L, 0.47 g/g	Keshav et al. 2016^143^
waste cotton materials	higher concentrated NaOH	0.35 g/g, 31.65%	SSSF	*S. cerevisiae* var. *ellipsoideus*	0.94 g/g glucose, 94.20%	Nikolić et al. 2017^144^
cotton stem waste	(1) mechanical; (2) enzymatic saccharification; (3) sequential alkali-acid	75–85%	SSF	*S. cerevisiae*	325 L/ton, 50%	Patel et al. 2017^145^
reed straw	unwashed + Tween 40 pretreatment	–	SSF (fed-batch)	*S. cerevisiae*	56.3 g/L, 69%	Lu et al. 2013^146^
raw corn straw	cold hydrolysis (commercial amylase and glucoamylase)	90% hydrolysis efficiency	modified SSF	*S. cerevisiae*	129.2 g/L (87.8%)	Šokarda Slavić et al. 2023^147^
soybean waste (okara)	autoclave pretreatment	120.5 g/L (70.5 g/L glucose and 50.0 g/L galactose)	enzymatic hydrolysis, fermentation	*S. cerevisiae*	59.1 g/L, 96.2%	Choi et al. 2015^148^
soybean residues	thermal acid hydrolysis (0.27 M, H_2_SO_4_, 121 °C, 60 min) + enzymatic saccharification	67.2 g/L (27.9 g/L glucose and 39.3 g/L galactose)	S-HF	*S. cerevisiae* adapted to a high % of galactose in a flask	33.9 g/L, 0.49 g/g glucose	Nguyen et al. 2018^149^
soybean hull hydrolysates	mechanical (milling, sieving)	0.96 g/L glucose, 1.11 g/L xylose, 0.43 g/L arabinose	(1) subcritical water hydrolysis; (2) 1:2 dilution; (3) fermentation supplemented with glucose (10 g/L)	*Wickerhamomyces* sp.	6.1 g/L	Vedovatto et al. 2021^150^
soybean hull	hydrothermal (imidazole, 120 °C, 1 h)	32.7 g/L of glucose, 9.4 g/L of xylose	enzymatic digestibility, fermentation	*S. cerevisiae*	12.9 g/L, 78.9%	Nishida et al. 2023^151^
orange peel waste	physical	–	ensiling for anaerobic digestion	leachate (10% w/w)	120 kg/ton	Fazzino et al. 2021^152^
pomegranate peel	hydrothermal	29.42 g/L	SSF	*S. cerevisiae*	12.9 g/L, 48.5%	Mazaheri et al. 2021 (153)
sugar cane straw	ball milling	30.5 g/L (29.8 g/L glucose and 0.7 g/L galactose), 78%	enzymatic hydrolysis and fermentation	*S. cerevisiae*	11.7 g/L, 91.8%	da Silva et al. 2010^154^
sugar cane bagasse	ball milling	25.7 g/L (24.9 g/L glucose and 0.8 g/L galactose), 77%	enzymatic hydrolysis and fermentation	*S. cerevisiae*	13.7 g/L, 89.8%	da Silva et al. 2010^154^
sugar cane bagasse	ball milling + hydrolysis	84.0% glucose	S-HF	*Pichia stipitis* BCC15191	8.4 g/L, 0.29 g/g	Buaban et al. 2010^155^
		70.4% xylose	SSF		8.0 g/L	
sugar cane bagasse	acid hydrolysis (0.5 M H_2_SO_4_)	glu: not detected, 1.44 g/L xylose	SSF	*S. cerevisiae* BTCC 3	2.43 g/L, 92.82%	Thontowi et al. 2018^156^
sugar cane bagasse	salt-alkali	–	SSF	*S. cerevisiae*	4.88 g/L, 0.49 g/g	Jugwanth et al. 2020^157^
sugar cane bagasse	solid-state fermentation (*Aspergillus tubingensis* NKBP-55)	22 g/L glucose, 16 g/L xylose	enzymatic hydrolysis and fermentation	*Candida shehatae* NCIM 3501	15.54 g/L, 77.9%	Prajapati et al. 2020^125^
sugar cane bagasse	physical, acid hydrolysis	50 g/L total sugar conc. hydrolysate	SSF	*P. stipitis*	10.62 g/L	Partovinia et al. 2022^158^
			combined hydrolysate fermentation		13.89 g/L	
sugar cane bagasse	acid–alkali	42 g/L glucose	S-HF	*Pichia occidentalis* AS.2	23.7 g/L, 21%	Saleh et al. 2022^159^
rice straw	acid–alkali	45 g/L glucose	S-HF	*P. occidentalis* AS.2	21.7 g/L, 24%	Saleh et al. 2022^159^
banana peduncle	acid–alkali	sugar concentration of 54.65 mg/g	S-SF	*K. marxianus*	25.39 g/L	Sathendra Elumalai et al. 2023^160^
pulp and paper sludge	steam explosion and NaOH treatment	79.56 g/L glucose, 8.65 g/L xylose	S-SSCF	*P. stipitis* NCIM 3499 and Baker’s yeast	42.34 g/L, 18%	Dey et al. 2021^161^
corn stover	unwashed + Tween 40 pretreatment	–	SSF (fed-batch)	*S. cerevisiae*	52.3 g/L, 71%	Lu et al. 2013^146^
corn cob (delignified residue)	–	98.16 g/L of glucose and 6.49 g/L of xylose	SSF	*S. cerevisiae*	75.07 g/L, yield: 89.38%	Lei et al. 2014^72^
corn stover	urea (4.87%) + steam explosion (1.22 MPa)	reducing sugars: 5.56–6.98 g/L after 72 h fermentation	enzymatic saccharification and fermentation	*S. cerevisiae*	EtOH conversion rate: 48.30%	Zhang et al. 2023^119^
corn straw	delignification (optimized water–ethanol system)	glucose yield: 19.80 g/L	SSSF	*S. cerevisiae*	titer >29.98 g/L	Ma et al. 2024^73^

a Separate hydrolysis and fermentation (S-HF); separate saccharification and fermentation (S-SF); simultaneous saccharification and fermentation (SSF); semisimultaneous saccharification and fermentation (SSSF); simultaneous saccharification and cofermentation (SSCF); semisimultaneous saccharification and cofermentation (S-SSCF); FPU, filter paper units, NaOH: sodium hydroxide; H_2_SO_4_: sulfuric acid; −: none/not applied; [TEA][HSO4]: ionic liquid triethylammonium hydrogen sulfate.

According to Khaire et al. (2021),^122^ sugar cane feedstock generates approximately 6000 L/hectare. Moreover, 1 ton of sugar cane generates 140 kg/t of bagasse and 250 kg of dry leaves, which can also be used in the fermentation process. Therefore, production is estimated to be 10 000 L/hectare, increasing if sugar cane residues are used for bioethanol synthesis. However, removing dry leaves and bagasse from the soil can harm the nutrition of future plantations; thus, it is not recommended to remove more than 50% of the soil for bioethanol synthesis. Fu et al. (2022)^123^ used sugar cane bagasse to produce bioethanol by SSF fermentation (*S. cerevisiae*, pH 4.8, 48 h, 37 °C) and achieved an efficiency conversion of 15.4% (w/w). Manmai et al. (2020)^124^ reused dry sugar cane leaves and reached 14% (w/w) of bioethanol.

On the other hand, Prajapati et al. 2020^125^ used cellulase and hemicellulase cocktail produced by *Aspergillus tubingensis* NKBP-55 to design an advantageous system for maximizing sugar cane bagasse feedstock for bioethanol using a glucose–xylose fermenting yeast *Candida shehatae* NCIM 3501, with efficient conversion (77.7%) of sugar cane bagasse hydrolysate to 2G ethanol. Therefore, bioethanol from residues of lignocellulosic materials presents the efficiency and reuse of residues to generate a product with high added value. Thus, studying the feasibility of other biomass residues can further increase bioethanol synthesis in the Brazilian energy matrix, reducing environmental damage and improving the management of industrial processes.^126−131^

### 4.1 Bioethanol Blending on Commercial Fuel Performance

Bioethanol shows a high compression ratio, reduced burning time, and less energy required for synthesis, making it superior to gasoline. The octane number serves to measure the quality of the fuel. A higher-octane rating can be achieved by high resistance to detonation^30,162^ and, hence, increases the compression ratio supported by the vehicle’s engine. An 87-octane fuel behaves like a mixture of 87% isooctane and 13% heptane.^48,163^ Ethanol is more environmentally friendly than fossil fuels once it emits less particulate matter and is produced from biomass residues. Table 3 shows the effect of adding bioethanol from sugar cane on the octane rating of base gasoline. Table 3 displays how the addition of bioethanol at different concentrations in the base gasoline affects the research octane number (RON) higher than the engine octane number (MON), increasing the fuel’s octane level in a concentration-dependent manner, generating a more efficient base gasoline.^49^ RON represents the combustion of engine fuel at low speeds and during acceleration. The MON describes the behavior of the fuel at high speeds and high loads.^164,165^ Thus, bioethanol has superior physicochemical characteristics compared to common ethanol, which favors its production and commercialization.^166−168^ Therefore, the replacement of common ethanol by bioethanol, or the mixture of these two substances, allows its application as a biofuel without losing the necessary characteristics of a typical fuel.

**Table 3:** Addition of Bioethanol in Base Fuel and Its Effect on Octane Rating^a^

			increase in octane with bioethanol							
composition of base gasoline			5% (v/v)		10% (v/v)		15% (v/v)		20% (v/v)	
aromatic	olefinics	saturated	MON	RON	MON	RON	MON	RON	MON	RON
50	15	35	0.1	0.7	0.3	1.4	0.5	2.2	0.6	2.9
25	25	50	0.4	1.0	0.9	2.1	1.3	3.1	1.8	4.1
15	12	73	1.8	2.3	3.5	4.4	5.1	6.6	6.6	8.6
11	7	82	2.4	2.8	4.6	5.5	6.8	8.1	8.8	10.6

a MON, engine octane number; RON, research octane number.

## 5 Future Opportunities with Crop Residues and Waste Produced in Brazil

Brazil is a promising country in implementing lignocellulosic biomass residues in the biofuel energy matrix. Raw materials can be analyzed to generate added value to waste obtained from industrial processes.^169^ In this Review, we identified potential in bioethanol production using other crop residues produced in Brazil responsible for billionaire economic movements in the national market:^169^ soybeans, rice, corn, cocoa, coffee (*Coffea arabica*),^148,170−172^ herbal cotton (*Gossypium hirsutum*),^173,174^ cassava (*Manihot esculenta*),^140,175^ tobacco (*Nicotiana tabacum*),^133^ and orange (*Citrus sinensis* L. Osbeck).^152^ These biomasses lead to an enormous generation of residues of interesting physicochemical characteristics potential in the industry, which are generally discarded and disregard a possible increase in added value.^60,125,176^ The production of biofuels from soy, corn, and sugar cane receives greater investment and optimization due to its greater abundance in Brazilian agribusiness. It generates a more significant amount of waste that could facilitate application in the synthesis of bioproducts of high economic and environmental value.^177^ Moreover, residues from coffee, cotton, rice, cassava, orange, and other fruits might present exciting characteristics for the synthesis of 2G ethanol.^178−183^ Aiming to present these crops as possible tools to encourage investments and studies around industrial applications for these residues, some studies were analyzed to examine the possibility of using residues from these lignocellulosic sources for application in ethanol synthesis.

Coffee (*C. arabica*) is an essential product in the national and international market, as Brazil is the world’s leading coffee exporter.^184^ Given its extensive production, we observe the enormous amount of waste generated along chain production such as coffee straws and grounds after consumption. The residues from this biomass are highly rich in organic material, caffeine, tannins, and polyphenols. The high amount of sugar in its composition, mainly mannose, makes this residue an excellent alternative for applications in the synthesis of 2G ethanol. However, mannose is found in galactomannans, which are highly hydrophilic and make pretreatment processes of residual biomass difficult.^170,171^ Nguyen et al. (2017)^171^ evaluated using coffee grounds with enzymatic pretreatment for synthesizing 2G ethanol using *S. cerevisiae KCTC 7906* as fermenter microorganisms. The study considered bioethanol synthesis by adding purification steps but pointed out the difficulty of pretreating mannose. Besides, the sugar content in coffee grounds residues may vary according to the coffee production chain, making process optimization difficult. Choi et al. (2012)^185^ also evaluated the use of coffee grounds for bioethanol synthesis, reaching a production of approximately 15.3 g/L. Despite this, there was difficulty in pretreating this residue, which can lead to higher costs for large-scale applications. However, applying the residue as a source for the fermentation and synthesis of 2G ethanol is still a promising novelty for industrial use.

Cotton generates a large amount of waste as stem, branches, bur, boll rinds, bracts, peduncle, roots, petioles, and leaf blades, opening a range of opportunities for its use in biorefineries, like 2G ethanol synthesis. Malik et al. (2021)^173^ analyzed cotton stems to synthesize bioethanol through physical and biological pretreatments to increase the contact of sugars with microorganisms for fermentation. The maximum efficiency of sugar utilization in the first fermentation cycle by immobilized yeasts was 94.1 and 90.4%, with the production of 0.46 and 0.44 g/g of bioethanol under chemical and biological pretreatment. Furthermore, the bioethanol yield was slightly sustained until the second cycle (0.38–0.40 g/g). However, bioethanol production continuously decreased in the third cycle and reached its lowest value in the fifth cycle. Besides stems, fibrous mixtures of cotton spinning residues can be applied to synthesize 2G ethanol. Ranjithkumar et al. (2022)^186^ evaluated the use of this residue in biofuel generation through the analysis of pretreatments and subsequent fermentation. Biological treatments for 14 days allowed a reduction of the cellulose crystallinity of approximately 80%, facilitating the fermentation process. Despite the success in synthesizing 2G ethanol, the production of this residue is much lower than crops, such as soy and sugar cane, which does not generate productive industrial competitiveness.

We observed that different types of residues from the most produced cultivars in Brazil can be evaluated and optimized for the synthesis of 2G ethanol. Table 2 shows some studies with residues generated by Brazilian cultivars prospected to contribute to enhancing bioethanol production in the country’s energy matrix.

Based on production, studies tend to use crops that commonly provide high-content residues. Therefore, not only by yield evaluation, the total amount of 2G bioethanol produced should be quantified based on the cultivated biomass and the annual production of its residues. In this way, relevant information will improve all processes and stages of biofuel production to create profit and environmentally friendly products.

The synthesis of 2G bioethanol comprises a complex and tunable production variable. The pretreatments mentioned in the work show a range of possibilities and combinations to increase the efficiency of the hydrolysis process and, consequently, the improvement in fermentation. ScCO_2_ can be combined with biological techniques since both apply mild temperature and pressure. In addition, a microbiological consortium might improve the hydrolysis of the lignocellulosic material once competition between microorganisms can generate more extracellular enzymes.

Another highly relevant strategy is the use of ion-dispersive hybrid units. The economic and employment benefits of maize ethanol produced from a second crop with soybeans in west-central Brazil can reduce greenhouse gas emissions compared with gasoline, which was suggested as biofuel feedstock.^187^ Thus, some studies evaluated the feasibility of producing hydrous ethanol from sugar cane bagasse in a conceptual hybrid unit under an energetically self-sufficient approach. Authors highlighted the high competitivity over other 2G bioethanol routes, achieving an ethanol yield of 330 L/BDT of biomass and an overall carbon conversion rate of 30%.^188^

## 6 Conclusions and Prospects

Corn meal and edible parts of sugar cane as a fermentative juice source for synthesizing 1G bioethanol are applied on a large scale in Brazil. Regarding 2G bioethanol, challenges must be overcome to increase its energy efficiency. Despite this, Brazil produces a range of lignocellulosic biomass from the agribusiness sector. Such residues and waste are even discarded in landfills. Therefore, the redirection of these residues for the synthesis of 2G bioethanol could significantly increase its commercial availability, added value, and environmentally friendly processes.

Several lignocellulosic residues produced in Brazil (i.e., from Amazonian babassu coconut starch, tobacco, cassava, orange, cotton, and potatoes) can benefit the energy matrix. Its combination with optimized processing parameters (pretreatment strategies) and fermenting (mixed culture) can increase the hydrolysis efficiency of lignocellulosic feedstock and then fermentation as well as the purification steps of produced ethanol. For example, the continuous insertion of substrates or other nutrients can favor contamination inside a reactor, causing a decrease in the bioethanol yield. The composition of babassu cake makes it an excellent raw material for solid-state fermentation in the bioethanol process, especially for the cultivation of filamentous fungi, enabling the production of complex multienzymes containing amylases, xylanases, cellulases, and proteases, which are capable of hydrolyzing starch granules crude.

However, researchers should direct future efforts to overcome challenges toward pretreatment strategies for lignocellulosic residues rich in aldohexoses series of carbohydrates (i.e., coffee straw and grounds after consumption, rich in mannose). After that, studying the feasibility of other biomass residues can further increase bioethanol synthesis in the fuel sector, reducing environmental damage and improving the management of industrial processes.

Other strategies based on the biorefinery concept of bringing 2G bioethanol into a hybrid unit should also be considered to produce different biofuels from food waste, as accomplished in Brazil with biomass gasification followed by syngas fermentation using acetogenic bacteria. In this context, lipids can be separated and converted into biodiesel by transesterification before the bioethanol production process; defatted lipid biomass can be converted into biomethane by anaerobic digestion; and dried residue from enzymatic hydrolysis can be transformed into other valued bioproducts for animal feed. Moreover, exploring the sustainability aspects of Brazilian agroindustrial waste valorization platforms through mixed culture fermentation and microbial consortia is crucial. A microbial consortium might improve the hydrolysis of the lignocellulosic material, once competition between microorganisms can generate more extracellular enzymes.

Finally, the industrial application of 2G bioethanol amplifies the commercialization of biofuels due to the high demand for environmentally friendly products with economic potential for the industry.
